# Use of Antithrombotics after Hemorrhagic Transformation in Acute Ischemic Stroke

**DOI:** 10.1371/journal.pone.0089798

**Published:** 2014-02-28

**Authors:** Joon-Tae Kim, Suk-Hee Heo, Man-Seok Park, Jane Chang, Kang-Ho Choi, Ki-Hyun Cho

**Affiliations:** 1 Department of Neurology, Cerebrovascular Center, Chonnam National University Hospital, Gwangju, Korea; 2 Biomedical Research Institute, Chonnam National University Hospital, Gwangju, Korea; 3 Department of Radiology, Chonnam National University Hwasun Hospital, Hwasun, Jeonnam, Korea; National University of Singapore, Singapore

## Abstract

**Backgrounds:**

There have been neither appropriate guidelines nor clinical studies about the use of antithrombotics after hemorrhagic transformation (HT). We sought to find whether the use of antithrombotics after hemorrhagic infarction might be associated with aggravation of HT and neurological deterioration.

**Methods:**

This retrospective study included prospectively registered consecutive patients with acute ischemic stroke and HT in our tertiary stroke center. We focused on the hemorrhagic infarction. Aggravation of HT was defined as either enlargement of the original HT or newly developed HT within the infarcted area by visual analysis. We analyzed relationships between antithrombotics and HT, and neurological deterioration after HT in patients with hemorrhagic infarction. In addition, we assessed composite outcomes including neurological deterioration, vascular events, and death at 1month after HT. We analyzed relationships between antithrombotics after discharge and composite outcomes within 1month after HT.

**Results:**

222 patients were finally analyzed. Of the 150 patients with hemorrhagic infarction, 75 (50.0%) were type 1. The use of warfarin after detection of hemorrhagic infarction more frequently increased aggravation of HT than did the use of antiplatelets (4 of 24 vs 3 of 69; *p* = 0.094), but neither warfarin nor antiplatelets caused more HT than no medication. In addition, the use of antithrombotics after hemorrhagic infarction was not significantly associated with neurological deterioration after HT. The frequency of composite events at 1months was significantly lower in patients treated with antithrombotics than those treated without (*p* = 0.041).

**Conclusion:**

In conclusion, the results of this study suggest that antithrombotics can safely be used after hemorrhagic infarction and may not be associated with neurological deterioration and aggravation of HT. Further studies are needed to confirm our results.

## Introduction

Hemorrhagic transformation (HT) is an important complication of ischemic stroke. In particular, HT might be one of the most problematic complications after thrombolysis. However, the clinical implications of HT are still unclear, and recent studies have shown that asymptomatic HT could be associated with poor outcomes [1], [2].

There have been neither appropriate guidelines nor clinical studies about the use of antithrombotics after HT. The American Heart Association/American Stroke Association's most recent guidelines suggest that HT within an ischemic stroke can be thought to have a different course and natural history from intracerebral hemorrhage (ICH) [3]. Therefore, the guidelines recommended that patients with cardioembolic stroke and hemorrhagic infarction continue anticoagulation treatment (Class IIb; Level of evidence C) [3]. However, many clinicians may still be reluctant to continue anticoagulation after HT.

There have been few studies on whether the use of antithrombotics after HT might enlarge hemorrhage in terms of size and extent, or aggravate clinical symptoms. A case series has shown that anticoagulation treatment after hemorrhagic infarction might be compatible with a stable clinical state and resolution of blood on follow-up imaging [4]. However, this should be cautiously interpreted due to the lack of large case series.

Therefore, we sought to investigate whether the use of antithrombotics after hemorrhagic infarction might be associated with aggravation of HT and neurological deterioration in acute ischemic stroke.

## Materials and Methods

### Subjects

This retrospective study included prospectively registered patients with acute ischemic stroke and HT in our tertiary stroke center who were admitted between August 2010 and March 2012. The patients complied from our stroke imaging protocol had ischemic lesions on diffusion-weighted imaging (DWI) and HT on brain images taken during admission. We excluded patients with (1) early loss of follow-up, including early death; (2) malignant infarction involving over two third of MCA territories; (3) bleeding complications such as hematuria; (4) history of recent bleeding or hemorrhagic disorders; and (4) brain surgery, such as decompression craniectomy.

### Ethics Statement

This study was approved by the Institutional Review Board (IRB) of Chonnam National University Hospital. Written informed consent was not obtained from participants because of the retrospective design of this study; therefore, the IRB of the hospital waived the need for written informed consent from participants.

### Demographic factors

Demographic and clinical data were collected from the prospective stroke registry. The following stroke risk factors were identified: age, sex, current cigarette smoking (cigarette smoking within the last 5 years), hypertension, diabetes mellitus, dyslipidemia, atrial fibrillation, and previous histories of stroke or transient ischemic attack. Baseline data collected from all patients included the National Institutes of Health Stroke Scales (NIHSS) scores and onset-to-visit times.

### Imaging protocol

According to our stroke imaging protocol, patients underwent emergency MRI immediately after admission. The MRI protocol consisted of DWI, fluid-attenuated inversion recovery (FLAIR), gradient echo (GRE) imaging, and time-of-flight MR angiography (MRA) in sequence. Routine follow-up imaging was performed twice at 3 (±12 hours) and 5 (±12 hours) days (computed tomography [CT] angiography and DWI/GRE, respectively) after initial MRI. In addition, DWI/GRE was also performed if neurologic deterioration occurred. Another imaging protocol for thrombolysis included the following procedures: emergency CT, brain MRI immediately after intra-venous thrombolysis, follow-up DWI/GRE at 24 hours after thrombolysis and then routine follow-up protocol. In patients with hemorrhage, follow-up imaging studies were performed to examine the changes (resolution or aggravation) of the hemorrhage 3–5 days after HT detection.

### Image analysis

HT cases were categorized as hemorrhagic infarcts (type 1 or 2) or parenchymal hematomas (type 1 or 2) according to the modified definitions established by a previous study [5]. We used GRE imaging for categorization of HT (Figure S1). A hemorrhagic infarct was defined as a petechial hemorrhage within the infarction area but with no space-occupying effect, while a parenchymal hemorrhage was defined as blood clots with the space-occupying effect. Aggravation of HT was defined as either enlargement of the original HT or newly developed HT within the infarcted area by visual analysis.

### Clinical analysis

We assessed neurological statuses at admission and on each hospital day by using NIHSS scores. Although this study was retrospective, neurological deterioration was carefully assessed by frequent neurological examinations. Neurological deterioration (ND) after HT was defined as any neurological deterioration considered to be caused by aggravation of HT during admission periods. In addition, we assessed composite outcomes including neurological deterioration, vascular events, and death at 1 month (±7 days) after HT. ND was differently defined from early neurological deterioration in this study (END, END was usually defined as an increase in NIHSS scores by 2 or more points or the development of new neurological symptoms within 5 days of admission).

The timing of the use of antithrombotics was identified to investigate whether antithrombotics might be associated with aggravation of HT. In cases treated with thrombolysis, antithrombotics should be started 24 hours after thrombolysis, based on follow-up imaging results. Aspirin was used for antiplatelets (100 to 300 mg at physicians' discretion) and only warfarin (initially with bridging medication of aspirin) for anticoagulation. We also investigated antithrombotics both at discharge and at a follow-up of 1 month. We analyzed relationships between antithrombotics during admission and aggravation of HT/ND after HT in patients with hemorrhagic infarction. In addition, we analyzed relationships between antithrombotics after discharge and composite outcomes within 1month after HT.

### Statistical analysis

Data are presented as mean ± SD or the frequency of categorical variables. Categorical variables were analyzed using the χ2 test and Fisher's exact test when appropriate. Continuous variables were analyzed using the independent sample *t* test or the Mann–Whitney *U* test when appropriate. We compared the frequency of HT, ND after HT, and composite outcomes within 1 month after HT between users and non-users of antithrombotics after HT in patients with hemorrhagic infarction. In order to evaluate the use of antithrombotics as a predictor of various types of outcomes (aggravation of HT), the use of antithrombotics was adjusted by age, NIHSS scores, and thrombolysis, in 150 patients with hemorrhagic infarction (model 1 by age and initial NIHSS, and model 2 by age, initial NIHSS, and thrombolysis). Odds ratios (ORs) and 95% confidence intervals (CIs) were calculated. A *p* value of <0.05 was considered statistically significant. All statistical analyses were performed using SPSS for Windows version 17 (SPSS Inc, Chicago, IL, USA).

## Results

### General characteristics of subjects

A total of 311 patients with HT within the infarcted area were screened during the study period. Of these patients, 89 were excluded: 56 were excluded due to malignant infarction. 29 were excluded due to loss of follow-up imaging studies (by early death or early loss of follow-up), and 4 were excluded due to surgeries, such as decompression craniectomy and hematoma removal. Ultimately, 222 patients (143 men; mean age, 70.45±11.42 years) were analyzed. Thrombolysis was performed on 104 of the 222 patients. One-hundred and thirty-four patients had atrial fibrillation.

Twenty-four (10.8%) patients had HT within the infarct area on initial MRI. The mean time between HT onset and hospital visit time was more delayed in patients with HT on initial MRI than in those without (1024.70 min vs 391.79 min, *p*<0.001) (Table S1). Of the patients with HT on the initial imaging, 6 patients showed aggravation of HT, but none showed ND after HT.

Overall, patients with parenchymal hemorrhage (n = 72) had higher NIHSS scores than those with hemorrhagic infarction (n = 150) (11.0 vs 13.0, *p* = 0.002). Thrombolysis and END were more frequent in patients with parenchymal hemorrhage than in those with hemorrhagic infarction (Table 1).

**Table 1:** General characteristics of subjects with hemorrhagic transformation at initial MRI and all imaging.

	Patients with Hemorrhagic infarction			All patients with HT		
	HI-1 (N = 75)	HI-2 (N = 75)	P	HI (N = 150)	PH (N = 72)	p
Age (mean±SD)	71.17±10.89	71.13±12.40	0.276	71.15±11.63	69.01±10.90	0.192
Male (n, %)	50 (66.7)	49 (65.3)	>0.999	99(66.0)	44(61.1)	0.549
Risk factors (n, %)						
HTN	47 (62.7)	53 (70.7)	0.387	100 (66.7)	52 (72.2)	0.443
DM	28 (37.3)	17 (22.7)	0.074	45 (30.0)	19 (26.4)	0.637
Smoking	24 (32.0)	17 (22.7)	0.272	41(27.3)	20 (27.8)	>0.999
Dyslipidemia	20 (26.7)	19 (25.3)	>0.999	39 (26.0)	11 (15.3)	0.087
Previous stroke	10 (13.3)	11 (14.7)	>0.999	21(14.0)	9 (12.5)	0.836
Atrial fibrillation	46 (61.3)	47 (62.7)	>0.999	93(62.0)	41 (56.9)	0.558
Visit time after onset, min (mean±SD),	560.68±792.6	425.00±727.24	0.983	492.84±761.12	392.25±913.00	0.389
Baseline NIHSS (med, IQR)	9.0 (11.0)	11.0 (9.0)	0.089	11.0 (9.0)	13.0 (6.75)	0.002
Thrombolysis (n, %)			0.827			0.006
IV only	17 (22.7)	21 (28.0)		38 (25.3)	28 (38.9)	
IA or IV+IA	12 (16.0)	11 (14.7)		23 (15.3)	17 (23.6)	
First anti-thrombotics (before HT) (n, %)	N = 68	N = 65	0.167			<0.001
None	8 (11.8)	17 (26.2)		65 (43.3)	61 (84.7)	
Anti-platelet	241 (60.3)	31 (47.7)		52 (34.7)	9 (14.8)	
Anti-coagulation	119 (227.9)	17 (26.2)		33 (22.0)	2 (2.8)	
Anti-thrombotics after HT (n, %)			0.001			<0.001
None	15 (20.0)	35 (46.7)		50 (32.3)	68 (94.4)	
Anti-platelet	41 (54.7)	31 (41.2)		72 (48.0)	4 (5.6)	
Anti-coagulation	19 (25.3)	9 (12.0)		28 (18.7)	0	
Neurological deterioration after HT (n, %)	2 (2.7)	4 (5.3)	0.681	6 (4.0)	10 (13.9)	0.012
Aggravation of HT at FU imaging (n, %)	5 (6.7)	11 (14.7)	0.185	16 (10.7)	14 (19.4)	0.093
Composite events within 1month (n, %)	1 (1.3)	4 (5.3)	0.367	5 (3.3)	7 (10.8)*	0..047

HTN, hypertension; DM, diabetes mellitus; NIHSS, National Institutes of Health Stroke Scale; IV, intravenous; IA, intra-arterial; HT, hemorrhagic transformation; END, early neurological deterioration; FU, follow-up.

*N = 65 (due to loss of follow-up).

### Use of anti-thrombotics in patients with hemorrhagic infarction

Of the 150 patients with hemorrhagic infarction, 75 (50.0%) were of type 1. The characteristics of these 150 patients are shown in Table 1. Aggravation of HT (n = 15) was more frequently observed in type 2 than in type 1 (11 vs 5, *p* = 0.185), but the frequency of ND after HT was not significantly different between types 1 and 2 (4 vs 2, *p* = 0.681). Furthermore, ND after HT was not related to aggravation of HT (2 of 6). In the use of antithrombotics, the number of patients treated with ‘no antithrombotics’ were more frequent in type 2 than in type 1 and the number of patients treated with ‘warfarin’ were less frequent in type 2 than in type 1 (*p* for trends = 0.001). Changes in the use of antithrombotics after HT are shown in Figure S2 and S3. The use of warfarin after hemorrhagic infarction was more reduced in type 2 than in type 1. The use of antiplatelets was also more reduced after detection of type 2 (Figure S3) than in type 1 of hemorrhagic infarction. In addition, of the 93 patients with cardioembolism and hemorrhagic infarction, only 28 (30.1%) continued to use warfarin after hemorrhagic infarction. The use of antiplatelets was also reduced from 86.0% to 73.7% in patients with non-cardioembolism. At discharge, the use of antithrombotics was as follows: no antithrombotics in 27 patients (18.0%), antiplatelets in 89 patients (59.3%), and warfarin in 34 patients (22.7%) (Figure S2). The use of antithrombotics at discharge was maintained over the first month of follow-up.

### Aggravation of HT/ND after HT/composite outcomes at 1month and the use of antithrombotics in hemorrhagic infarction

**Figure 1:**
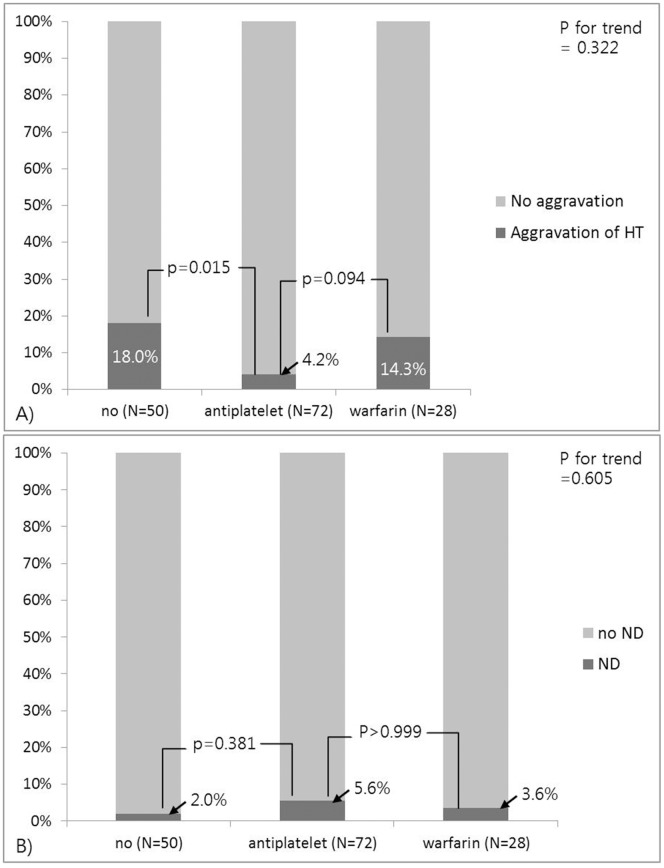
In hemorrhagic infarction (N = 150), a comparison of the development of HT aggravation (A) and neurological deterioration after HT (B) associated with the use of anti-thrombotic medication after HT. Abbreviations: HI, hemorrhagic infarction; HT, hemorrhagic transformation; ND, neurological deterioration after HT; END, early neurological deterioration.

The use of warfarin after detection of hemorrhagic infarction more frequently increased aggravation of HT than did the use of antiplatelets (4 of 24 vs 3 of 69; *p* = 0.094), but neither warfarin nor antiplatelets caused more HT than no medication (Figure 1-A). In addition, the use of antithrombotics after hemorrhagic infarction was not significantly associated with ND after HT (Figure 1-B). Aggravation of HT was related to ND after HT in only 2 of the 16 patients (*p* = 0.124) and was not related to transition to parenchymal hemorrhage from hemorrhagic infarction. Multivariate logistic regression analysis revealed that the use of antiplatelets after hemorrhagic infarction was negatively associated with aggravation of HT (Model 2: OR 0.153; 95% CI 0.036–0.657, *p* = 0.012), whereas the use of warfarin was not (Table 2). Furthermore, the use of antithrombotics (warfarin and antiplatelets) after hemorrhagic infarction was not associated with ND after HT (Table 3).

**Table 2:** The associations between use of antithrombotics with aggravation of hemorrhagic transformation by multivariate logistic regression analysis in patients with hemorrhagic infarction (N = 150).

	Model 1	p	Model 2	p
Anti-thrombotics before HT		0.467		0.475
No med	Ref		Ref	
Antiplatelet	0.489 (0.117–2.049)	0.328	0.508 (0.120–2.147)	0.357
Warfarin	0.965 (0.199–4.679)	0.965	1.021 (0.206–5.053)	0.980
Anti-thrombotics after HT		0.038		0.041
No med	Ref		Ref	
Antiplatelet	0.150 (0.035–0.642)	0.011	0.153 (0.036–0.657)	0.012
Warfarin	0.517 (0.124–2.152)	0.365	1.186 (0.375–3.755)	0.771

Aggravation of HT; Model 1: Adjusted by age and initial NIHSS. Model 2: Adjusted by age, initial NIHSS, and thrombolysis.

END; adjusted by age, initial NIHSS and thrombolysis.

**Table 3:** The associations between use of antithrombotics with ND after HT by multivariate logistic regression analysis in patients with hemorrhagic infarction (N = 150).

	Model 1	p	Model 2	p
Use of antithrombotics after HT	2.332 (0.246–22.150)	0.461	2.503 (0.264–23.759)	0. 424
Initial NIHSS scores	0.964 (0.827–1.124)	0.639	0.938 (0.787–1.117)	0.470
Age	1.062 (0.963–1.171)	0.230	1.065 (0.965–1.175)	0.214
Thrombolysis	NA		2.228 (0.353–14.056)	0.394

Model 1: Adjusted by age and initial NIHSS. Model 2: Adjusted by age, initial NIHSS, and thrombolysis.

The frequency of composite events at 1months was significantly lower in patients treated with antithrombotics than those treated without (1.6% vs 11.1%, *p* = 0.041). In addition, by multivariate logistic regression, composite events at 1 month after HT were significantly associated with the use of antithrombotics after discharge (Model 1: OR 0.132; 95% CI 0.020–0.893; *p* = 0.038) (Table 4).

**Table 4:** The associations between use of antithrombotics with composite outcomes at 1 month by multivariate logistic regression analysis in patients with hemorrhagic infarction (N = 150).

	Model 1	p	Model 2	p
Use of antithrombotics after DC	0.132 (0.020–0.893)	0.038	0.128 (0.018–0.908)	0.040
Initial NIHSS scores	0.986 (0.835–1.165)	0.889	0.999 (0.847–1.177)	0.989
Age	1.019 (0.927–1.120)	0.700	1.016 (0.925–1.116)	0.743
Thrombolysis	NA		0.316 (0.033–3.061)	0.320

Model 1: Adjusted by age and initial NIHSS. Model 2: Adjusted by age, initial NIHSS, and thrombolysis.

DC; discharge.

### Aggravation of HT and the use of antithrombotics based on clinical situation in patients with hemorrhagic infarction

In patients treated with thrombolysis (n = 106), HT was mostly observed on 24-h GRE images obtained after thrombolysis (n = 96, 90.6%). Of the 106 thrombolysed patients, 61 developed hemorrhagic infarction. Of these 61 patients, 3 (9.4%) with type 2 hemorrhagic infarction developed ND after HT. There were no significant differences in aggravation of HT between types 1 and 2 (n = 3, 10.3% vs n = 4, 12.5%). After thrombolysis, 19 patients (31.1%) used no antithrombotics, 32 patients (52.5%) used antiplatelets, and 10 patients (16.4%) used warfarin, while after detection of hemorrhagic infarction, 1 user of warfarin and 6 users of antiplatelets stopped the use of antithrombotics. Aggravation of HT after the first use of antithrombotics was detected in 4 antiplatelet users, 2 warfarin users, and 1 patient who was not treated with antithrombotics (12.5% vs 20.0% vs 5.3%), without statistical significance. Therefore, the frequencies of aggravation HT after hemorrhagic infarction were 15.4% (4/26), 11.5% (3/26), and 0% (0/9) in non-users of antithrombotic, antiplatelet, or warfarin after HT, respectively (*p* for trends = 0.246), with no statistical significance (Table 5).

**Table 5:** Types of hemorrhagic infarction based on thrombolysis and clinical and imaging changes associated with use of antithrombotics.

	Before hemorrhagic infarction			After hemorrhagic infarction		
**Thrombolysis (N = 61)**	No med	Antiplatlelet	Warfarin	No med	Antiplatelet	Warfarin
A) Hemorrhagic infarction type 1 (N = 29)	n = 7	n = 18	n = 4	n = 8	n = 15	n = 6
HT aggravation	1 (14.3)	2 (11.1)	0	2 (25.0)	1 (6.7)	0
ND after HT	0	0	0	0	0	0
B) Hemorrhagic infarction type 2 (N = 32)	n = 12	n = 14	n = 6	n = 18	n = 11	n = 3
HT aggravation	0	2 (14.3)	2 (33.3)	2 (11.1)	2 (18.2)	0
ND after HT	0	2 (14.3)	1 (16.7)	1 (5.6)	2 (18.2)	0
**Non-thrombolysis (N = 89)**						
A) Hemorrhagic infarction type 1 (N = 46)	n = 2	n = 26	n = 18	n = 7	n = 26	n = 13
HT aggravation	0	0	2 (11.1)	0	0	2 (15.4)
ND after HT	0	1 (3.8)	1 (5.6)	0	1 (3.8)	1 (7.7)
B) Hemorrhagic infarction type-2 (N = 43)	n = 10	n = 22	n = 11	n = 17	n = 20	n = 6
HT aggravation	3 (30.0)	2 (9.1)	2 (18.2)	5 (29.4)	0	2 (33.3)
ND after HT	0	1 (4.5)	0	0	1 (5.0)	0

HT, hemorrhagic transformation; ND, neurological deterioration.

Eighty-nine of the 116 patients who did not undergo thrombolysis showed hemorrhagic infarction; only 33 patients (39.3%) showed hemorrhagic infarction on follow-up imaging within 2 days of initial MRI and the rest on follow-up imaging 3 days after initial MRI. Aggravation of HT was observed in 2 (4.3%) of the 46 patients with type 1 hemorrhagic infarction and 7 (16.3%) of the 43 patients with type 2 hemorrhagic infarction (*p* = 0.083). ND after HT was observed in 3 patients (2 patients in types 1 and 1 patient in type 2) (Table 5). Four (13.8%) of the 29 patients who used warfarin before HT showed aggravation of HT, all of whom continued to use warfarin after HT. However, ND after HT did not correlate with aggravation of HT (p = 0.277).

## Discussion

Our study showed that although physicians may be reluctant to use antithrombotics after HT, even after hemorrhagic infarction, the use of antithrombotics after hemorrhagic infarction was not associated with ND after HT or aggravation of HT. In addition, although aggravation of HT was more frequently observed in patients with type 2 of hemorrhagic infarction than in those with type 1, the use of antithrombotics after hemorrhagic infarction was not associated with ND after HT or significant deleterious changes in the hemorrhagic infarction. Therefore, our study suggested that the use of antithrombotics after hemorrhagic infarction might be reasonable for prevention of subsequent ischemic stroke. However, because this was a retrospective study, further studies with a prospective design are needed to confirm our results.

In our study, about 66% of all patients used antithrombotics after hemorrhagic infarction, but only 30% of patients who required warfarin used warfarin after hemorrhagic infarction. The use of antiplatelets was reduced to about 73% in patients who had hemorrhagic infarction and non-cardioembolic stroke. Physicians were also reluctant to use antiplatelets after hemorrhagic infarction. Recent guidelines from the American Heart Association/American Stroke Association recommended that patients who need warfarin should continue to receive warfarin treatment regardless of hemorrhagic infarction (Class IIb; Level of evidence C) [3]. However, there is still a lack of evidence for this because only small case series have been conducted so far [4]. Our study, albeit a single-center retrospective study, demonstrates important factors physicians should consider when they detect HT in patients with acute ischemic stroke and the value of weighing the harms over the benefits of antithrombotics after HT.

Although the use of antiplatelets after HT has not been recommended in any trials, antiplatelet therapy should ideally be initiated within 24 to 48 hours of stroke onset [6]. Because patients with acute ischemic stroke do not generally receive follow-up imaging, asymptomatic HT may not easily be detected in actual practice. However, we were readily able to find asymptomatic HT by routine repetitive follow-up imaging studies in acute clinical setting. Our study showed that the acute use of antiplatelets after hemorrhagic infarction may be relatively safe. ND after HT was uncommon in the use of antithrombotics after hemorrhagic infarction. These results suggest that there may be no firm basis for physicians to reject to use antithrombotics after hemorrhagic infarction.

Asymptomatic HT is mainly considered to be an epiphenomenon of reperfusion into ischemic tissue without any clinical impact [7], [8]. Only parenchymal hemorrhage (type 2) significantly increased the risk of early clinical deterioration and worse outcomes. Other types of HT, such as types 1 and 2 of hemorrhagic infarction, were not associated with deterioration after HT [9], [10]. However, early anticoagulation after ischemic stroke could increase the risk of HT and is not recommended in the very early stage after ischemic stroke for patients with moderate to severe strokes [6]. Therefore, our results suggest that the cautious use of antithrombotics (continuation or temporarily discontinuation of the antithrombotics), was not related to the aggravation of HT and ND after HT even when hemorrhagic infarction is present on follow-up imaging. Discontinuing or continuing antithrombotics for a few days followed by repeat imaging to evaluate the stability of hemorrhagic infarction may then allow for the safe continuation of treatment. In our study, only 30% of patients with hemorrhagic infarction continue to use warfarin in acute ischemic stroke. The use of antithrombotics after hemorrhagic infarction can be supported by previous studies and ours.

We assessed the influence of the use of antithrombotics on composite events over early periods of follow-up. Even without aggravation of HT on follow-up imaging, 18% of patients did not use antithrombotics at the time of discharge. This may be due to higher risk of events in patients treated with no antithrombotics than in those treated with antithrombotics in the early periods. Although the use of antithrombotics after HT may raise concern about aggravation of HT, the risk of events may increase in patients treated without antithrombotics after HT. Therefore, the results of our study suggest that meticulous care may be needed over early periods after HT.

The results of this study are subject to some limitations. First, being a single-center, retrospective study that evaluated consecutive patients with HT according to our stroke protocol, further studies with a prospective design are needed to confirm our results. In particular, we could not confirm the efficacy of antithrombotics in patients with hemorrhagic infarction because they were neither randomly selected nor blinded to antithrombotics. However, in order to minimize a selection bias, we only analyzed patients with hemorrhagic infarction. In addition, since the number of warfarin users after hemorrhagic infarction was small, relationships between the use of warfarin after hemorrhagic infarction and aggravation of HT/ND after HT need to be further investigated through prospective studies. Furthermore, the effects of antithrombotic agents, either antiplatelet or anticoagulant, need at least 3 to 5 days to reach the optimal level, if not given loading dose initially. In our study, MR images were followed at 3–5 days after HT detection which may not be adequate time window to evaluate the drug effect on hematoma aggravation or other possible complications. We evaluated composite events at 1month after HT in order to overcome this limitation. Factors related to HT have been shown to be various clinical conditions, such as chronic kidney disease, high blood pressure, and infarct size. However, because we included only patients with HT in acute ischemic stroke in this study, these factors were excluded from the study. Finally, we excluded patients with malignant infarction over two-thirds of hemisphere. The risk of HT might be high in these patients. However, we considered that malignant infarction per se affect the safety and ND and thus should be excluded in this study.

In conclusion, the results of this study suggest that antithrombotics can safely be used after hemorrhagic infarction and may not be associated with ND after HT and aggravation of HT. However, many physicians are still reluctant to use antithrombotics when HT is detected by imaging, even in hemorrhagic infarction. Further studies are needed to confirm our results.

## Supporting Information

Table S1 **Characteristics of subjects with hemorrhagic transformation on initial imaging.**
(DOCX)

Figure S1 **Types of hemorrhagic transformation according to the gradient echo imaging, modified by ECASS definition.** A) Hemorrhagic infarction type 1. There are small petechiae along the margin of the infarct. B) Hemorrhagic infarction type 2. There are confluent petechiae within the infarcted area, but without space-occupying effect. C) Parenchymal hemorrhage type 1. There is a hematoma in 30% or less of infarcted area with some slight space-occupying effect. D) Parenchymal hematoma type 2. There is a dense hematoma over 30% of infarcted area with substantial space occupying effect, or as any hemorrhagic lesion outside infarcted area.
(TIF)

Figure S2 **Changes in the use of anti-thrombotics before and after hemorrhagic infarction and at a follow-up of 1 month in patients with hemorrhagic infarction.**
(TIF)

Figure S3 **Changes in the use of anti-thrombotics before and after hemorrhagic infarction.** Even after hemorrhagic infarction, use of anti-thrombotics decreased.
(TIF)

## References

[1] Park JH, Ko Y, Kim WJ, Jang MS, Yang MH, et al (2012) Is asymptomatic hemorrhagic transformation really innocuous? Neurology 78: 421–426. 22282643 10.1212/WNL.0b013e318245d22c

[2] Kent DM, Hinchey J, Price LL, Levine SR, Selker HP (2004) In acute ischemic stroke, are asymptomatic intracranial hemorrhages clinically innocuous? Stroke 35: 1141–1146. 15087567 10.1161/01.STR.0000125712.02090.6e

[3] Furie KL, Kasner SE, Adams RJ, Albers GW, Bush RL, et al (2011) Guidelines for the prevention of stroke in patients with stroke or transient ischemic attack: a guideline for healthcare professionals from the american heart association/american stroke association. Stroke 42: 227–276. 20966421 10.1161/STR.0b013e3181f7d043

[4] Pessin MS, Estol CJ, Lafranchise F, Caplan LR (1993) Safety of anticoagulation after hemorrhagic infarction. Neurology 43: 1298–1303. 8327127 10.1212/wnl.43.7.1298

[5] Hacke W, Kaste M, Fieschi C, von Kummer R, Davalos A, et al (1998) Randomised double-blind placebo-controlled trial of thrombolytic therapy with intravenous alteplase in acute ischaemic stroke (ECASS II). Second European-Australasian Acute Stroke Study Investigators. Lancet 352: 1245–1251. 9788453 10.1016/s0140-6736(98)08020-9

[6] Adams HP Jr, del Zoppo G, Alberts MJ, Bhatt DL, Brass L, et al (2007) Guidelines for the early management of adults with ischemic stroke: a guideline from the American Heart Association/American Stroke Association Stroke Council, Clinical Cardiology Council, Cardiovascular Radiology and Intervention Council, and the Atherosclerotic Peripheral Vascular Disease and Quality of Care Outcomes in Research Interdisciplinary Working Groups: the American Academy of Neurology affirms the value of this guideline as an educational tool for neurologists. Stroke 38: 1655–1711. 17431204 10.1161/STROKEAHA.107.181486

[7] Fiehler J, Remmele C, Kucinski T, Rosenkranz M, Thomalla G, et al (2005) Reperfusion after severe local perfusion deficit precedes hemorrhagic transformation: an MRI study in acute stroke patients. Cerebrovasc Dis 19: 117–124. 15640606 10.1159/000083180

[8] Molina CA, Alvarez-Sabin J, Montaner J, Abilleira S, Arenillas JF, et al (2002) Thrombolysis-related hemorrhagic infarction: a marker of early reperfusion, reduced infarct size, and improved outcome in patients with proximal middle cerebral artery occlusion. Stroke 33: 1551–1556. 12052990 10.1161/01.str.0000016323.13456.e5

[9] Berger C, Fiorelli M, Steiner T, Schabitz WR, Bozzao L, et al (2001) Hemorrhagic transformation of ischemic brain tissue: asymptomatic or symptomatic? Stroke 32: 1330–1335. 11387495 10.1161/01.str.32.6.1330

[10] Kablau M, Kreisel SH, Sauer T, Binder J, Szabo K, et al (2011) Predictors and early outcome of hemorrhagic transformation after acute ischemic stroke. Cerebrovasc Dis 32: 334–341. 21921596 10.1159/000331702

