# Supplementary material for: Use of Antithrombotics after Hemorrhagic Transformation in Acute Ischemic Stroke
Source: PLoS One. 2014 Feb 28;9(2):e89798. doi: 10.1371/journal.pone.0089798 (PMC3938534; doi:10.1371/journal.pone.0089798)
Supplement: Table S1 — Characteristics of subjects with hemorrhagic transformation on initial imaging. (DOCX) [file pone.0089798.s001.docx]

Table S1. Characteristics of subjects with hemorrhagic transformation on initial imaging

|  | On the initial imaging | | P |
| --- | --- | --- | --- |
|  | No HT  (N=150) | HT  (N=23) |  |
| Age | 70.80±11.9 | 67.5±10.5 | 0.215 |
| Male (n, %) | 94 (62.7) | 19 (82.6) | 0.097 |
| Risk factors  HTN  DM  Smoking  Dyslipidemia  Previous stroke  Atrial fibrillation | 104 (69.3)  38 (25.3)  44 (29.3)  39 (26.0)  16 (10.7)  91 (60.7) | 16 (69.6)  9 (39.1)  5 (21.7)  5 (21.7)  3 (13.0)  12 (52.2) | >0.999  0.208  0.620  0.800  0.722  0.497 |
| Visit time after onset (mean, min) | 335.3±634.5 | 1006.6±1426.7 | <0.001 |
| Baseline NIHSS (med, IQR) | 12.0 (9.0) | 8.0 (11.0) | 0.015 |
| Anti-thrombotics after HT  None  Anti-platelet  Anti-coagulation | 97 (64.7)  36 (24.0)  17 (11.3) | 13 (56.5)  7 (30.4)  3 (13.0) | 0.527 |
| Early neurological deterioration  END  Severe END | 38 (25.3)  27 (18.0) | 1 (4.3)  1 (4.3) | 0.029  0.131 |
| Aggravation of at FU imaging | 21 (14.1) | 5 (21.7) | 0.352 |

HTN, hypertension; DM, diabetes mellitus; NIHSS, National Institutes of Health Stroke Scale; HT, hemorrhagic transformation; END, early neurological deterioration; FU, follow-up.
