# Supplementary figures and images for: Use of Antithrombotics after Hemorrhagic Transformation in Acute Ischemic Stroke
Source: PLoS One. 2014 Feb 28;9(2):e89798. doi: 10.1371/journal.pone.0089798 (PMC3938534; doi:10.1371/journal.pone.0089798)

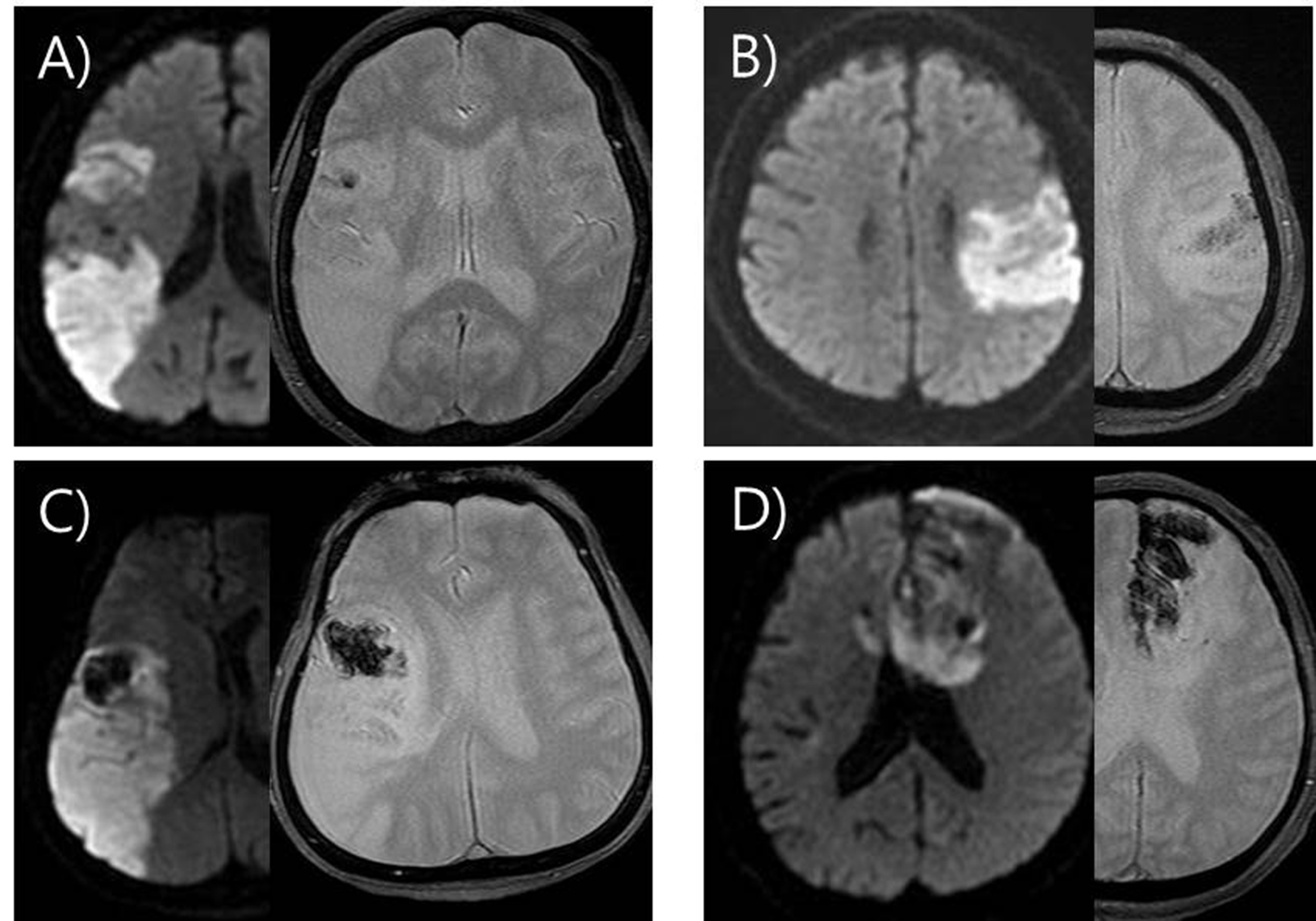

Supplement: Figure S1 — Types of hemorrhagic transformation according to the gradient echo imaging, modified by ECASS definition. A) Hemorrhagic infarction type 1. There are small petechiae along the margin of the infarct. B) Hemorrhagic infarction type 2. There are confluent petechiae within the infarcted area, but without space-occupying effect. C) Parenchymal hemorrhage type 1. There is a hematoma in 30% or less of infarcted area with some slight space-occupying effect. D) Parenchymal hematoma type 2. There is a dense hematoma over 30% of infarcted area with substantial space occupying effect, or as any hemorrhagic lesion outside infarcted area. (TIF) [file pone.0089798.s002.tif]

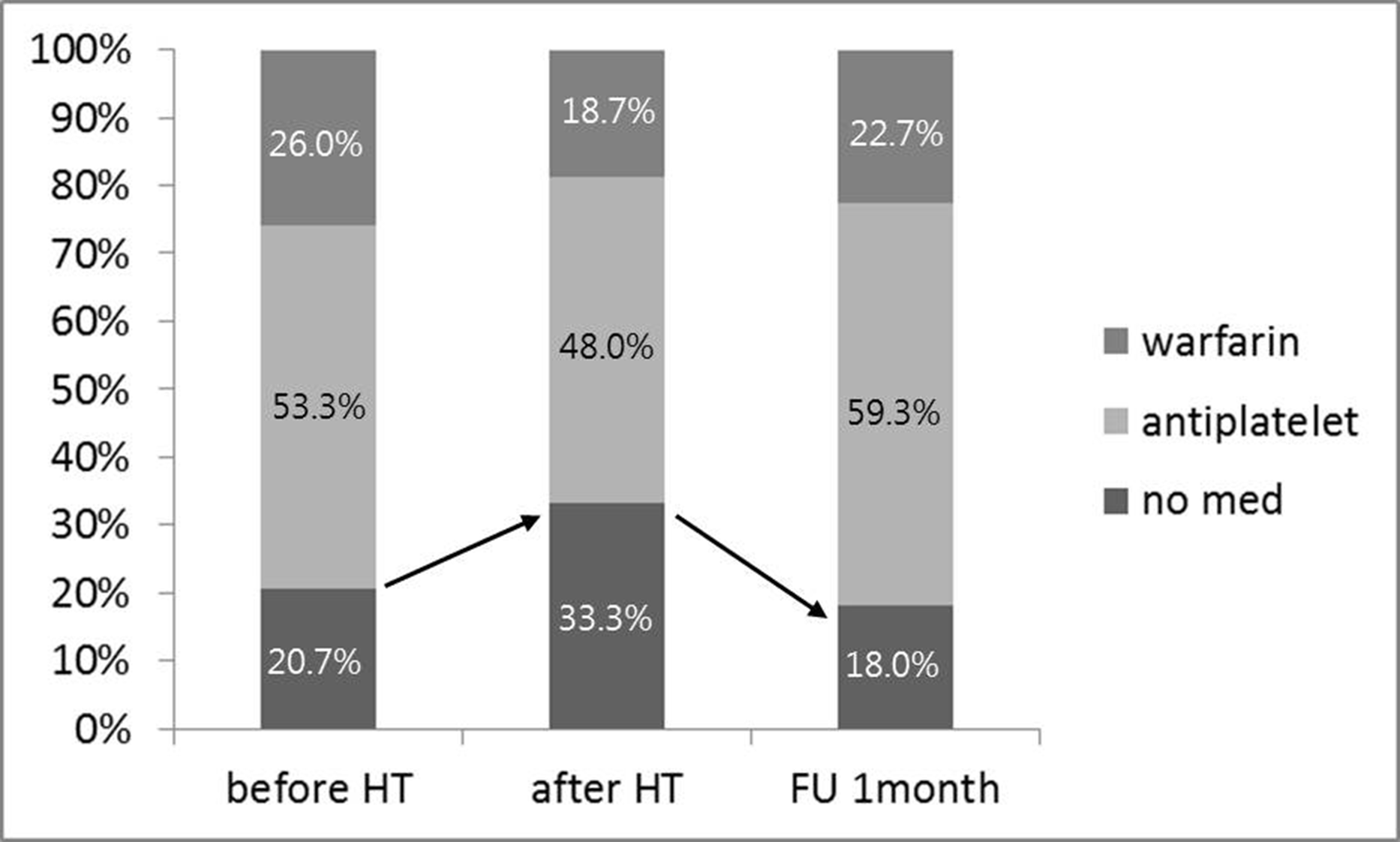

Supplement: Figure S2 — Changes in the use of anti-thrombotics before and after hemorrhagic infarction and at a follow-up of 1 month in patients with hemorrhagic infarction. (TIF) [file pone.0089798.s003.tif]

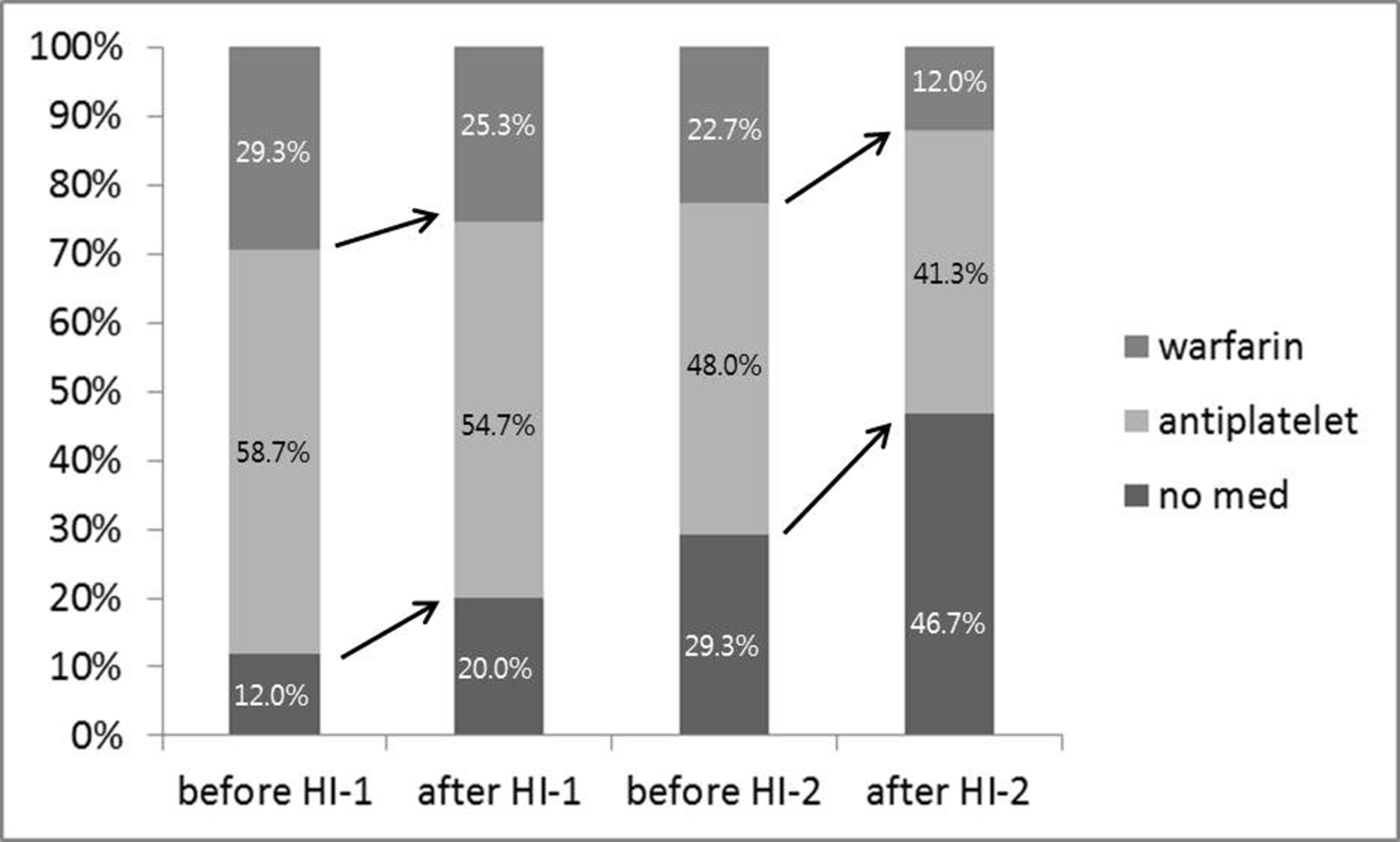

Supplement: Figure S3 — Changes in the use of anti-thrombotics before and after hemorrhagic infarction. Even after hemorrhagic infarction, use of anti-thrombotics decreased. (TIF) [file pone.0089798.s004.tif]
